# Organic semiconductor-incorporated two-dimensional halide perovskites

**DOI:** 10.1093/nsr/nwab111

**Published:** 2021-06-24

**Authors:** Yao Gao, Letian Dou

**Affiliations:** Davidson School of Chemical Engineering, Purdue University, USA; Davidson School of Chemical Engineering, Purdue University, USA; Birck Nanotechnology Center, Purdue University, USA

## Abstract

Organic semiconductor-incorporated halide perovskites (OSiP) have emerged as a promising new family of 2D hybrid materials with exceptional structural tunability, optical and electronic properties, and environmental stability.

Recently, the search for novel two-dimensional (2D) organic-inorganic halide perovskite materials has come to a renaissance. There is significant interest not only in terms of their intriguing optoelectronic properties, but also their structural diversity and improved stability. The 2D perovskite structure can be visualized as atomically thin slabs cut from 3D parent structures along different crystal directions that are sandwiched between two layers of large organic cations. The bulky organic cations/ligands play a governing role in 2D structure formation via suppression of the out-of-plane growth and promotion of in-plane growth. Furthermore, these bulky hydrophobic organic cations provide protection to the vulnerable halide perovskite lattice.

**Figure 1.**
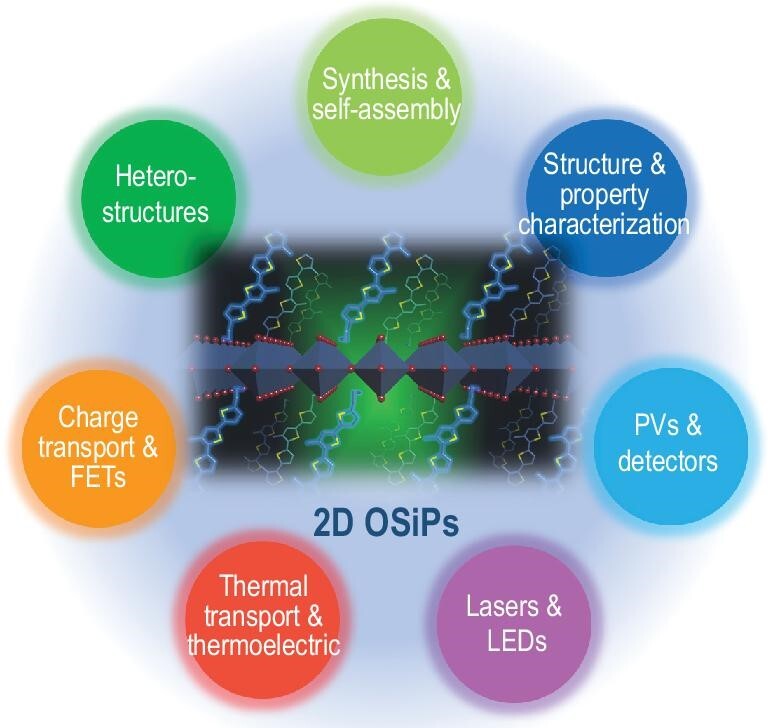
Research scope of the emerging two-dimensional organic semiconductor-incorporated perovskites (OSiPs).

Currently, most 2D halide perovskites employ simple organic cations, for example butyl ammonium (BA) or phenylethyl ammonium (PEA), which function solely as the insulating barriers in the formed 2D structure. Semiconducting organic cations could play a more active role in tuning overall optical and electronic properties, but this type of hybrid structure is less well explored. Here, we call this type of perovskite an ‘organic semiconductor-incorporated perovskite’, or simply ‘OSiP’ (Fig. 1). One of the first single crystalline OSiPs reported by Mitzi *et al.* in 1999, incorporates a quaterthiophene derivative with an amino group on both ends, in which the quaterthiophene unit leads to quenching of photoluminescence of the perovskite layer [1]. Another example of an OSiP was reported in the same year incorporating a pyrene unit with tunable phosphorescence properties [2]. Synthesizing such complex structures has been challenging. During the past two decades, there have been sparse efforts and only a few π-conjugated organic moieties have been reported. Recently, we expanded the library of OSiPs using oligothiophene-based monoammonium cations [3]. Through judicious molecular design, a series of π-conjugated ligands with tunable energy levels and band gaps have been synthesized and successfully incorporated into the 2D perovskite lattice. Complete band alignment tuning from type-I to type-II to reverse type-I and to reverse type-II was demonstrated. These organic-inorganic heterostructures provide a new platform on which to study the ultrafast charge and energy transfer across the heterogeneous interfaces using femtosecond spectroscopy tools [4]. Furthermore, the conjugated ligands not only encapsulate the perovskite layer but also provide a driving force for lattice stabilization, resulting in significantly improved chemical and thermal stability. This work paves the way for fabrication of high-performance semiconductor heterostructures and devices.

Epitaxial growth provides another dimensionality for tuning and enhancing the optoelectronic properties of crystalline semiconductor materials in general. However, this has been challenging in halide perovskites because of rapid ion migration and interdiffusion. Notably, a series of highly stable and tunable epitaxial lateral heterostructures have been fabricated in 2D perovskites recently [5]. By incorporating conjugated organic ligands, it was shown that the ion interdiffusion across the heterojunction interface can be substantially suppressed. Near atomically sharp interfaces are thus obtained and further deciphered by low-dose aberration-corrected high-resolution transmission electron microscopy. Using corresponding conjugated organic ligands, larger vacancy formation energies of 2D perovskites and the reduced heterostructure disorder were confirmed in molecular dynamics simulations. Additionally, vertical heterojunctions between different phase-pure 2D perovskites were successfully constructed recently [6,7]. How the organic ligand affects anionic diffusion at the ligand-perovskite interface was investigated [6]. Intriguingly, it was demonstrated that anion diffusion across these vertical heterostructures did not follow the classical Fickian diffusion of their 3D counterparts featuring continuous concentration profile evolution. Instead, a ‘quantized’ layer by layer diffusion model governed by local free energy minimum and ion-blocking effect from the organic ligands is proposed to describe the anionic migration behavior. Surprisingly, anionic interdiffusion coefficients for 2D perovskites with short aliphatic organic ligands (e.g. BA) are not much smaller than those of their 3D counterparts, suggesting the short aliphatic ligands are not effective in blocking the anion diffusion. On the other hand, bulkier and more rigid organic conjugated ligands were found to inhibit halide interdiffusion much more effectively with estimated diffusion coefficient less than 10^–20^ m^2^/s. These works provide important insights regarding ion diffusion in the halide perovskite solids. The realization of both lateral and vertical 2D halide perovskite heterostructures offers a diverse platform for revealing the interplay between different 2D perovskites (e.g. strain effects and charge-transfer excitons) and corresponding optoelectronic device applications (e.g. diodes, photodetectors and electronic-photonic integrated circuits).

The renascent OSiP also have tremendous potential in device applications, but again, this is not well studied. For example, the organic semiconductor ligands are qualified as electronically active components that enable better charge transport in out-of-plane directions. Enhanced out-of-plane conductivity has recently been demonstrated by incorporating a series of acene compounds. The (pyrene-*O*-propyl-NH_3_)_2_PbI_4_ displayed improved photovoltaic performance and out-of-plane conductivity orders of magnitude higher than that of (BA)_2_PbI_4_ [8]. The semiconducting organic ligand can also serve as a multifunctional interface modifier inserted between the light-harvesting perovskite film and the hole transporting layer, effectively passivating the surface defects, extracting holes from the perovskite, facilitating charge transfer between the perovskite and hole transporting layer, and suppressing ion migration and improving device stability [9]. 2D halide perovskites are promising for light-emitting diode (LED) applications because of their natural quantum well characteristics. Their single crystals have been shown to have high photoluminescence quantum efficiencies and ultra-narrow emission bandwidth. However, limited by extremely poor stability and inefficient charge injection, a reliable electrically driven LED device has not been realized. An Sn (II)-based 2D perovskite quantum well using molecularly tailored organic semiconductor layers for efficient and stable LEDs was reported recently [10]. Benefiting from the small bandgap organic semiconductor, charge injection and transport have been improved, and ion migration has also been greatly inhibited. Thus, a hybrid quantum well lead-free LED device with superior performance has been demonstrated featuring a pure red emission, a peak external quantum efficiency up to 3.3%, a maximum luminance of 3466 cd m^–2^ and an operational stability of over 150 h.

A field-effect transistor (FET) is usually employed to evaluate in-plane charge transport in 2D perovskites. The parallel growth orientation properties enable 2D perovskites to be used effectively in these lateral-structure devices. Our recent work demonstrated the first application of OSiP in the FET device, and found that the quaterthiophene-based organic ligand (4Tm) significantly strengthened the ambient and thermal stability of Sn perovskite-based FET [11]. Compared with the benchmark material (PEA)_2_SnI_4_ which could degrade quickly even being stored in nitrogen glovebox, (4Tm)_2_SnI_4_ displayed a high retention in FET performance even after a month. Besides, the (4Tm)_2_SnI_4_ FET device exhibited reduced hysteresis and near ten-fold enhancement in hole mobility (2.32 cm^2^ V^–1^s^–1^). The improved hole mobility can be ascribed to the active role that the 4Tm ligand plays in the hole injection process, thanks to favorable energy level alignment. It was also proposed that 4Tm ligand could further inhibit ion migration better than the PEA ligand, which agrees well with our interdiffusion studies in the heterostructures. It is worth mentioning that 2D perovskite thin film morphology with larger grain texture was obtained using this organic conjugated ligand, pointing towards potential manipulation of OSiP crystallization by modulating the organic moieties. Finally, the organic building blocks can be modified through post-treatment such as doping. In a recent work, it was proven that the polydiacetylene moieties incorporated in 2D halide perovskite can be p-doped, which forms radicals and leads to drastic improvement in light absorption and electrical conductivity [12].

The OSiPs have emerged as an exciting family of semiconducting materials. Through combination of organic semiconducting building blocks, their optical and electronic properties have been greatly improved. Meanwhile, the rigid and bulky organic semiconducting moieties have proven useful in improving environmental stability as well as inhibiting intrinsic ion migration. Enhanced stability and reduced hysteresis in optoelectronic devices have also been obtained. Although the vast choices of organic semiconducting spacers sandwiching the perovskite layers provides huge possibilities for these hybrid combinations, incorporation of these large conjugated organic moieties in spatially confined 2D perovskite is still challenging. Hence, rational design and functionalization of the organic components in OSiP are needed. A deeper fundamental understanding of how molecular design influences the overall crystal structure, thin film morphology and optoelectronic properties of the OSiP is needed as well. Nevertheless, we envision that many novel structures can be demonstrated and exciting breakthroughs in fundamental science as well as device applications of OSiP will be achieved in the near future, with great efforts from the community.
